# Potency of Xanthone Derivatives from *Garcinia mangostana* L. for COVID-19 Treatment through Angiotensin-Converting Enzyme 2 and Main Protease Blockade: A Computational Study

**DOI:** 10.3390/molecules28135187

**Published:** 2023-07-04

**Authors:** Cecep Suhandi, Siti Sarah Alfathonah, Aliya Nur Hasanah

**Affiliations:** 1 Department Pharmaceutics and Pharmaceutical Technology, Faculty of Pharmacy, Universitas Padjadjaran, Sumedang 45363, Indonesia; 2 Department Pharmaceutical Analysis and Medicinal Chemistry, Faculty of Pharmacy, Universitas Padjadjaran, Sumedang 45363, Indonesia

**Keywords:** xanthone, ACE2, Mpro, *Garcinia mangostana* L., garcinone B, molecular docking

## Abstract

ACE2 and Mpro in the pathology of SARS-CoV-2 show great potential in developing COVID-19 drugs as therapeutic targets, due to their roles as the “gate” of viral entry and viral reproduction. Of the many potential compounds for ACE2 and Mpro inhibition, α-mangostin is a promising candidate. Unfortunately, the potential of α-mangostin as a secondary metabolite with the anti-SARS-CoV-2 activity is hindered due to its low solubility in water. Other xanthone isolates, which also possess the xanthone core structure like α-mangostin, are predicted to be potential alternatives to α-mangostin in COVID-19 treatment, addressing the low drug-likeness of α-mangostin. This study aims to assess the potential of xanthone derivative compounds in the pericarp of mangosteen (*Garcinia mangostana* L.) through computational study. The study was conducted through screening activity using molecular docking study, drug-likeness prediction using Lipinski’s rule of five filtration, pharmacokinetic and toxicity prediction to evaluate the safety profile, and molecular dynamic study to evaluate the stability of formed interactions. The research results showed that there were 11 compounds with high potential to inhibit ACE2 and 12 compounds to inhibit Mpro. However, only garcinone B, in addition to being indicated as active, also possesses a drug-likeness, pharmacokinetic, and toxicity profile that was suitable. The molecular dynamic study exhibited proper stability interaction between garcinone B with ACE2 and Mpro. Therefore, garcinone B, as a xanthone derivative isolate compound, has promising potential for further study as a COVID-19 treatment as an ACE2 and Mpro inhibitor.

## 1. Introduction

The need to improve the quality of COVID-19 (coronavirus disease 2019) treatment cannot be ruled out. The ongoing global pandemic has caused widespread devastation, resulting in immense suffering and an unfathomable loss of lives [1]. Although vaccination efforts have offered some optimism, the emergence of new variants and breakthrough infections underscores the urgency to develop more efficacious treatments [2,3]. The significance of improved medicines for COVID-19 is multifaceted. They can effectively mitigate the severity of symptoms, thereby alleviating the strain on healthcare systems and averting overwhelming surges in hospitalizations [4,5]. Consequently, advanced therapeutics can specifically target distinct stages of the disease, yielding superior outcomes for patients in varying phases of infection [6]. Furthermore, the implementation of effective antiviral drugs can contribute to shorter recovery periods, facilitating an expedited return to normalcy for individuals and mitigating the socio-economic ramifications of the pandemic [7].

The available medications for COVID-19 offer certain treatment possibilities, but the need for more effective and safe medicine to combat the disease is evident. Currently, specific antiviral drugs like remdesivir have received emergency use authorization for particular cases [8,9]. These medications work by impeding the replication of the virus within the body, potentially reducing the duration of illness [10]. Other studies have also shown that remdesivir correlates well with the inhibition of Mpro (main protease), which results in the inhibition of virus release from host cells [11,12]. Additionally, chloroquine has shown promise in treating SARS-CoV-2’s infections by inhibiting the ACE2 receptor, which serves as a gateway for the virus to enter pulmonary host cells [13,14]. Blocking these receptors prevents the virus from further infecting the human body [15]. However, while these treatments have demonstrated some effectiveness, they are not universally successful, and their impact on patient outcomes is still under research. Although chloroquine was found to be effective in inhibiting the virus through ACE2 blockade in preclinical studies [16], clinical studies did not show great relevance for both the prevention and cure of COVID-19 patients [17,18]. Giving chloroquine to COVID-19 patients did not correlate with symptom improvement, viral clearance, or reduced mortality [18]. Moreover, these medical options also have limitations. Chloroquine, in particular, is not recommended for treating COVID-19 patients due to its tendency to cause higher toxic effects compared to the obtained effectiveness [19,20]. Therefore, enhanced medicine for COVID-19 is essential to address the limitations of existing options, improve patient outcomes, and ultimately bring an end to the devastating consequences of the pandemic.

Among the many available options, the utilization of the potential of natural substances from various plant sources has become highly sought after. Secondary bioactive compounds from plants are known to have advantages, as they not only have potential pharmacological activities but are also considered safer than synthetic chemical compounds [21]. One that has garnered significant attention in the development of COVID-19 therapy is α-mangostin [22,23,24]. α-mangostin is a xanthone derivative compound sourced from the pericarp of *Garcinia mangostana* L. [25,26]. It is known for its ability to prevent the entrance of SARS-CoV-2 into host cells through ACE2 and Mpro inhibition [23,27].

**Figure 1:**
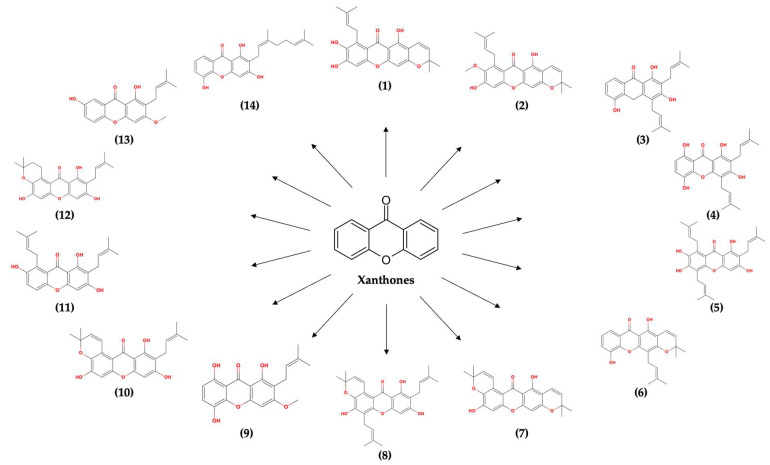
Fourteen xanthone isolates obtained from the pericarp of *Garcinia mangostana* L.

However, the potential of α-mangostin tends to be hindered due to its unfavorable physicochemical properties. Its low solubility in water makes it difficult to formulate into drug preparations, especially for oral administration, which is commonly preferred by patients [28,29]. Interestingly, the potential of other isolated xanthone derivative compounds from the pericarp of *Garcinia mangostana* L. has not been further explored for their potency in combating SARS-CoV-2 and their compatibility to be formulated as oral drug preparations. To date, 14 other xanthone derivative compounds have been identified in the pericarp of the mangosteen fruit, including 7-*O*-demethyl mangostanin, mangostanin, 8-deoxygartanin, gartanin, garcinone E, trapezifolixanthone, padiaxanthone, tovophyllin A, 1,5,8-Trihydroxy-3-methoxy-2-prenylxanthone, garcinone B, 1,7-dihydroxy-2-(3-methylbut-2-enyl)-3-methoxyxanthone, mangostenone D, mangostinone, and 1,7-dihydroxy-2-(3-methylbut-2-enyl)-3-methoxyxanthone, respectively, as isolated compounds numbered (**1**) to (**14**), as illustrated in Figure 1 [30].

Therefore, this research aims to serve as a preliminary study to investigate the potential of xanthone derivative compounds in the pericarp of Mangosteen fruit as anti-SARS-CoV-2 agents. The potential of these compounds is assessed based on their ability to inhibit ACE2 and Mpro, while their suitability for formulation into drugs is evaluated through Lipinski’s rule of five compatibility studies and pharmacokinetic and toxicity profiles. Additionally, molecular dynamics studies are conducted to observe the interaction stability of potential compounds with the target receptor in SARS-CoV-2.

## 2. Results

### 2.1. Molecular Docking Simulation

The molecular docking study of xanthone isolates towards ACE2 is presented in Table 1. Almost all isolates have lower binding energy compared to chloroquine, remdesivir, and α-mangostin, with the exception of isolate 2 (mangostanin), isolate 5 (garcinone E), and isolate 8 (tovophyllin A). The inhibition constant of each isolate is well correlated with their binding energy. The lower the binding energy, the lower the inhibition constant obtained [31]. Furthermore, all of the isolates demonstrated a proper mode of interaction with ACE2, represented by key amino acid residues of ACE2 which were interacted with the isolate. The key amino acids include Gln24, Thr27, Asp30, His34, Glu35, Tyr41, Gln42, Met82, and Lys353 [32,33]. Overall molecular docking results indicated that all of the xanthone isolates, except isolate 2 (mangostanin), isolate 5 (garcinone E), and isolate 8 (tovophyllin A), have great potency as an ACE2 inhibitor.

In molecular docking simulations against Mpro, 12 isolates showed lower binding energy than chloroquine, remdesivir, and α-mangostin. Only isolate 2 (mangostanin) and isolate 11 (1,7-dihydroxy-2-(3-methylbut-2-enyl)-3-methoxyxanthone) did not exhibit similar results. As shown in Table 2, all xanthone isolates demonstrated appropriate interaction modes by interacting with key amino acid residues in Mpro, including His41, Cys145, and Glu166 [27]. Therefore, only isolate 2 (mangostanin) and isolate 11 (1,7-dihydroxy-2-(3-methylbut-2-enyl)-3-methoxyxanthone) have a lower potential for Mpro inhibition compared to chloroquine, remdesivir, and α-mangostin.

### 2.2. Lipinski’s Rule of Five Filtration

This filtration was aimed to highlight the most suitable isolate for oral drug administration. The compatibility of each xanthone isolate regarding the rule is recapped in Table 3. Filtration of all isolates implied that isolate 2 (mangostanin), isolate 3 (8-deoxygartanin), isolate 4 (gartanin), isolate 6 (trapezifolixanthone), isolate 8 (tovophyllin A), isolate 10 (garcinone B), and isolate 12 (mangostenone D) were suitable as drugs for oral administration.

### 2.3. Pharmacokinetic Profile and Toxicity Prediction

The overall pharmacokinetic profiles of all xanthone isolates are recapped in Table 4. The absorption parameter is represented by human intestinal absorption (HIA) and caco-2 cell permeability. Distribution is denoted by protein plasma binding (PPB) and blood–brain barrier (BBB). Metabolism is symbolized by cytochrome P450 (CYP450) inhibition or substrate. Toxicity is characterized by carcinogenicity and mutagenicity (Ames test) [34]. The overall results indicated that only isolate 10 (garcinone B) fulfilled all the pharmacokinetic profiles and toxicity attribute requirements.

### 2.4. Molecular Dynamic Simulation

**Figure 2:**
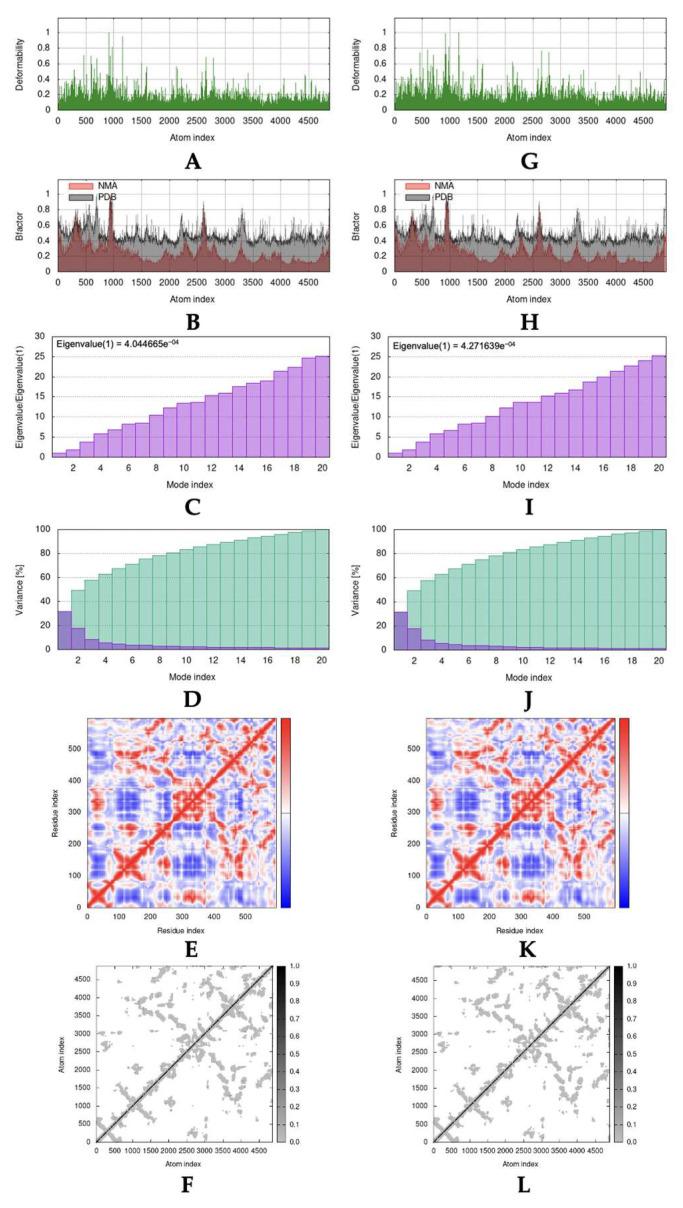
The parameters representing molecular dynamic simulation results of isolate 10 (garcinone B) interacting with ACE2: (**A**) deformability, (**B**) B-factor, and (**C**) eigenvalue; (**D**) variance, (**E**) co-variance map, and (**F**) elastic network of native ACE2; (**G**) deformability, (**H**) B-factor, and (**I**) eigenvalue; (**J**) variance, (**K**) co-variance map, and (**L**) elastic network of ACE2 complexed with garcinone B.

**Figure 3:**
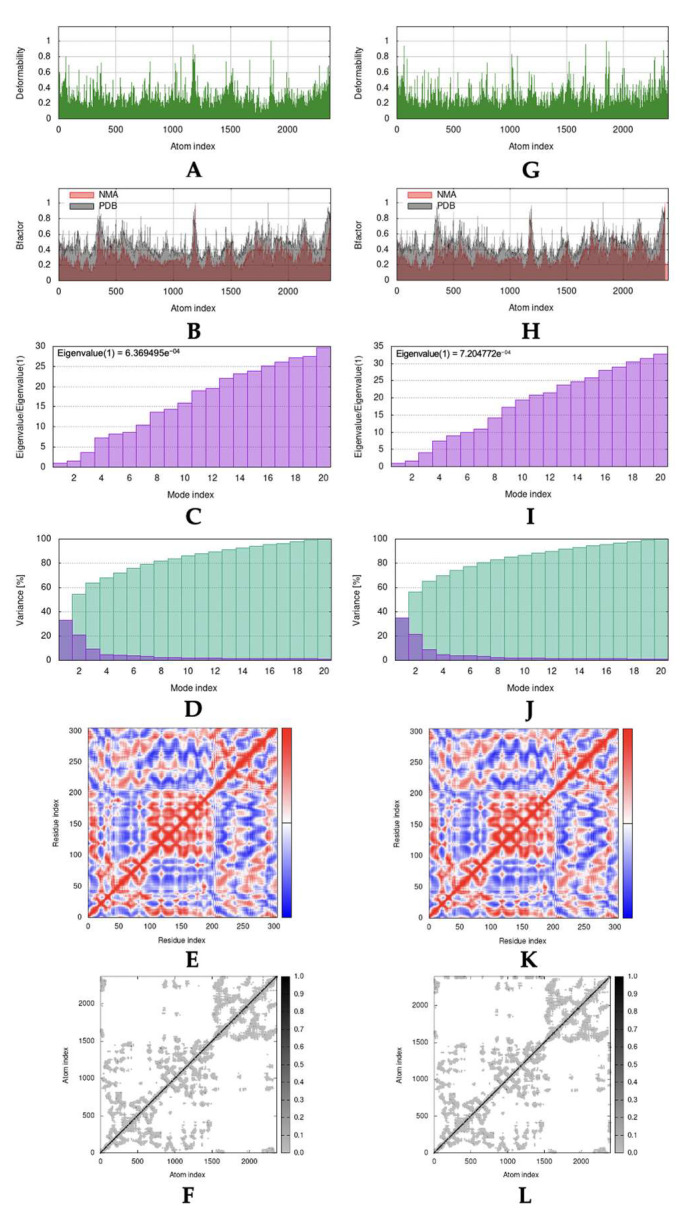
The parameters representing molecular dynamic simulation results of isolate 10 (garcinone B) interacting with Mpro: (**A**) deformability, (**B**) B-factor, and (**C**) eigenvalue; (**D**) variance, (**E**) co-variance map, and (**F**) elastic network of native ACE2; (**G**) deformability, (**H**) B-factor, and (**I**) eigenvalue; (**J**) variance, (**K**) co-variance map, and (**L**) elastic network of Mpro complexed with garcinone B.

As the result of molecular docking, Lipinski’s rule of five filtration, and pharmacokinetic profile and toxicity prediction, it was revealed that garcinone B was the most potential and suitable compound. Molecular dynamic simulation was only accomplished toward a complex formed between garcinone B with ACE2 and Mpro. Figure 2 and Figure 3 summarized results that exhibited stability interaction based on normal mode analysis (NMA) of garcinone B with ACE2 and Mpro, compared with the native form of ACE2 and Mpro. The deformability of native ACE2, ACE2-garcinone B complex, native Mpro, and Mpro-garcinone B complex was represented in Figure 2A,G and Figure 3A,G, respectively [35]. The B-factors between NMA mobility and PDB fields of native ACE2, ACE2-garcinone B complex, native Mpro, and Mpro-garcinone B complex were presented in Figure 2B,H and Figure 3B,H, respectively [36]. Eigenvalues that correspond to motion stiffness of the native ACE2, ACE2-garcinone B complex, native Mpro, and Mpro-garcinone B complex structures were depicted in Figure 2C,I and Figure 3C,I, respectively [37]. Inversion of eigenvalues resulting in variance of each individual (purple bars) and cumulative (green bars) of native ACE2, ACE2-garcinone B complex, native Mpro, and Mpro-garcinone B complex was illustrated in Figure 2D,J and Figure 3D,J, respectively [37]. Co-variance map analysis (Figure 2E,K and Figure 3E,K) indicated the correlation, lack of correlation, and anti-correlation between pairs of amino acid residues [37]. Finally, the motion stiffness of native ACE2, ACE2-garcinone B complex, native Mpro, and Mpro-garcinone B complex structures were also explained through dot mapping on elastic networks (Figure 2F,L and Figure 3F,L) [35]. Visually, the overall data indicate a similarity in stability between the native protein structure and the complex. However, the higher eigenvalue obtained in the complex structures (4.271639e^−04^ and 7.204772e^−04^) compared to the native receptors (4.044665e^−04^ and 6.369495e^−04^) suggests that the complexes have higher stability. These results indicate that the interaction between garcinone B and the target receptors is stable.

## 3. Discussion

### 3.1. Molecular Docking Simulation

Molecular docking studies on xanthone derivative compounds as anti-SARS-CoV-2 agents have been successfully conducted using AutoDock 4.2.6. The simulation of the anti-SARS-CoV-2 activity of xanthone isolates was analyzed based on their ability to inhibit the ACE2 receptor and Mpro. ACE2 is abundantly found on the surface of alveolar cells and plays a crucial role in the regulation of the circulatory system [38]. In alveolar cells, ACE2 functions as a barrier defense against cell injury and inflammation [39]. In the vascular system, ACE2 is known to break down angiotensin II into angiotensin-(1,7), thereby dilating blood vessels and lowering blood pressure [40]. In the pathology of COVID-19 infection, ACE2 serves as the gateway for the virus to infect cells [41]. The spike protein present in SARS-CoV-2 matches the conformation of ACE2, acting like a key and lock mechanism [42]. Thus, inhibition of ACE2 can impact the prevention of cell entry due to the inability to interact properly with ACE2 [43]. The mechanism of ACE2 inhibition has shown great potential in the treatment of SARS-CoV-2 infection. It has been proven in research that the use of ACE2 antibodies (hACE2.16) can prevent viral entry in various types of variants [44]. Furthermore, Mpro also plays a crucial role in preventing the spread of the virus that has successfully infected host cells. Mpro is involved in cleaving the polyprotein produced from viral RNA transcription for the maturation of new viruses [45]. Inhibition of Mpro results in the failure of virus reproduction and prevents the formation of new viruses that could potentially infect other healthy cells. Thus, the search for small molecules capable of inhibiting ACE2 and Mpro could be the promising choice, as well as overcoming the cost hurdles in developing monoclonal antibodies.

**Figure 4:**
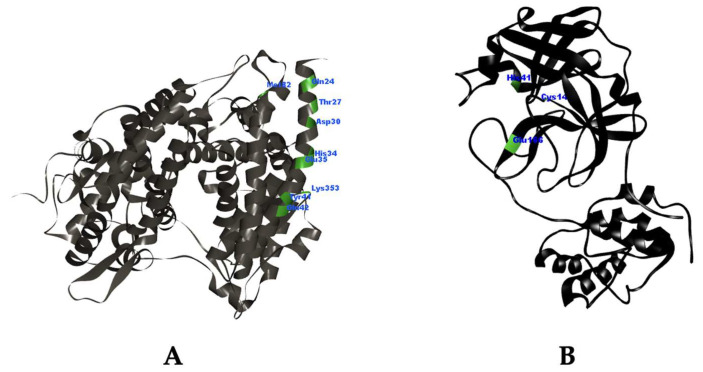
3D visualization of ACE2 extracted from complex (PDB ID: 6M0J) (**A**) and Mpro extracted from complex (PDB ID: 6LU7) (**B**).

In this study, the three-dimensional model of ACE2 was obtained from the crystal structure of ACE2 interacting with the binding region of SARS-CoV-2’s spike protein in the Protein Data Bank (PDB) with the code ID: 6M0J, while the three-dimensional structure of Mpro was obtained from the crystal structure of Mpro interacting with the compound N3 in the PDB with the code ID: 6LU7. The 3D structures of ACE2 and Mpro were depicted in Figure 4A,B, respectively. The crystal complex structure of ACE2 and Mpro was elucidated through X-ray diffraction with a resolution value of 2.45 Å and 2.16 Å, respectively [46,47]. The selection of this crystal structure was based on the requirement of good resolution quality, which is estimated to be <2.5 Å [48]. In addition, chloroquine and remdesivir were utilized as comparators or standard drugs in this study. The selection of chloroquine and remdesivir as standard drugs is based on scientific evidence stating that only chloroquine and remdesivir, as marketed drugs, have been proven to be active as an ACE2 and Mpro inhibitor, respectively, in COVID-19 treatment [12,16].

Based on the results of molecular docking simulations, a total of 11 isolates showed better potential for ACE2 inhibition activity compared to chloroquine, remdesivir, and α-mangostin, while 12 isolates were more potential as Mpro inhibitor than all standards used. This implication is stated based on the lower values of the lowest binding energy (ΔG) and inhibition constant (Ki) obtained. A smaller ΔG value indicates that the intermolecular interaction between the ligand and the receptor occurs more spontaneously [49]. The Ki value correlates well with the prediction of the minimal dose required to produce an inhibitory effect on the receptor [31]. Additionally, the mode of interaction with amino acid residues on the target receptor is crucial [50]. Ligand interactions with amino acid residues Gln24, Thr27, Asp30, His34, Glu35, Tyr41, Gln42, Met82, and Lys353 in ACE2 result in the inhibition of SARS-CoV-2’s spike protein binding [32,33]. Moreover, interactions towards His41, Cys145, and Glu166 are well-correlated with the catalytic inhibition of Mpro [27]. All isolates showed predicted interaction modes that could bind to both key ACE2 and Mpro amino acids, potentially inhibiting the binding by the spike protein and inhibiting polyprotein cleavage in viral maturation processes. Among all isolates, isolate 13 (mangostinone) exhibited the best effectiveness potential, as evidenced by the lowest ΔG value and the lowest Ki value, correlating with the lowest effective dose. However, when selecting active drug compounds, not only effectiveness but also considerations of formulation design compatibility and safety should be deliberated.

### 3.2. Lipinski’s Rule of Five Filtration

As an oral drug is considered the most preferred dosage form due to its convenience for patients, Lipinski’s rule of five filtration can predict the drug-likeness of organic compounds that can provide good bioavailability and permeability [51]. It is based on physicochemical parameters, including molecular weight, the number of hydrogen bond donors, hydrogen bond acceptors, and the LogP value [52]. The molecular weight correlates with the molecule’s capability to pass through pores in the cell membrane, while hydrogen bond donors and acceptors, as well as LogP, are related to the compound’s polarity properties, enabling good solubility in water and facilitating membrane penetration [51,52]. Half of the analyzed isolates (isolate 1, 5, 7, 9, 11, 13, and 14) showed one violation in the LogP value exceeding five, while the other seven isolates did not exhibit any violations. Violations against the LogP value exceeding 5 result in the compound being lipophilic, leading to low solubility in water and consequently impacting the low bioavailability of the compound in plasma.

### 3.3. Pharmacokinetic Profile and Toxicity Prediction

The pharmacokinetic predictions indicated that all isolates have good absorptivity through the human intestine (indicated by HIA > 0.9) [53]. Based on the predicted membrane permeability in Caco-2 cells, only isolates 3, 7, 9, and 11 showed high permeability (>0.7), while the others exhibited moderate permeability (0.04–0.7) [54]. In terms of distribution, isolates 1, 3, 5, 11, and 14 showed strong binding ability to plasma proteins (>0.9), while the others have weak binding to plasma proteins [55]. Furthermore, all isolates were predicted to penetrate the blood–brain barrier (BBB) at a moderate level (0.04–0.7) [56]. All isolates have the ability to inhibit CYP450 enzymes and do not act as substrates for these enzymes. This revealed that all isolates can potentially affect the metabolism of other drugs that are metabolized by CYP450, while the isolates themselves were not metabolized by CYP450 enzymes. These findings suggest that the administration of isolates as drugs should not be given simultaneously with drugs that act as CYP450 substrates [57]. Another interesting finding in the toxicity prediction was that only isolate 10 (garcinone B) is both non-carcinogenic and non-mutagenic.

### 3.4. Molecular Dynamic Simulation

**Figure 5:**
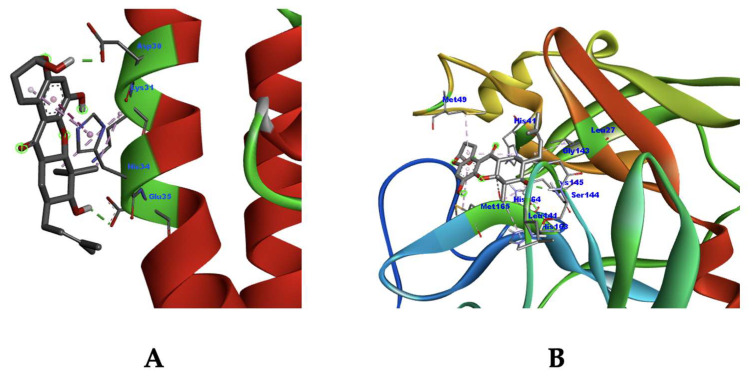
3D visual interaction of isolate 10 (garcinone B) with ACE2 (**A**) and Mpro (**B**).

Based on the overall tests in the molecular docking study, Lipinski’s rule of five filtration, and pharmacokinetic and toxicity profile prediction, only isolate 10, garcinone B, fulfilled all the acceptance criteria. Although garcinone B did not provide the best ΔG value, this compound has suitable interaction modes as an ACE2 and Mpro inhibitor (Figure 5) and has the best safety profile among the other isolates. As supplementation, other studies have demonstrated the potential pharmacologic activity of this compound as an anti-inflammatory agent [58]. Garcinone B was known to have anti-inflammatory abilities through the inhibition of COX enzymes, resulting in the inhibition of prostaglandin release. Furthermore, the inhibition of necrosis factor (NF-κB) expression also contributes to its immunomodulatory effect [58]. These facts have positive implications for its potential as a COVID-19 drug since COVID-19 patients also display a hyperinflammatory condition [59].

**Figure 6:**
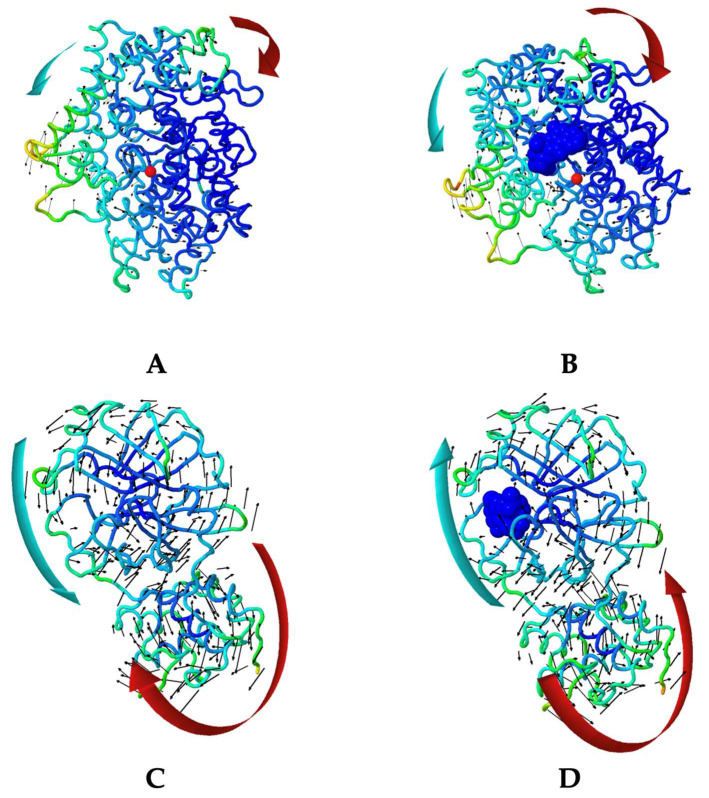
Molecular dynamic visual presentation of native ACE2 (**A**), complex between isolate 10 (garcinone B) and ACE2 (**B**), native Mpro (**C**), and complex between isolate 10 (garcinone B) and Mpro (**D**).

Molecular dynamic studies on the interaction complex between garcinone B (isolate 10) with ACE2 and Mpro showed good interaction stability. An overview of the motion pattern of native ACE2, Mpro, and the complex between garcinone B with ACE2 and Mpro was presented in Figure 6A,B, respectively. Visually, the deformability of ACE2 has not changed significantly, as can be seen from the identical “hinge region” peaks with relatively the same intensity. Similar findings were also shown for other parameters, including b-factor, variance, co-variance map, and elastic network. Different findings that stand out can be seen from the eigenvalues obtained, where each complex has a higher acquisition of eigenvalues (4.271639e^−04^ and 7.204772e^−04^) than each native receptor (4.044665e^−04^ and 6.369495e^−04^). A higher eigenvalue correlates with a higher energy requirement to initiate the deformation of the formed structure [60]. Thus, the eigenvalue analysis indicates that the intermolecular interactions that occur between garcinone B with ACE2 and Mpro were stable. These results were also comparable to a similar study by Abdelli et al., which used isothymol, another potential ACE2 inhibitor compound [36]. The eigenvalue gain for the isothymol-ACE2 complex is lower, which indicates a higher deformability that tends to be less stable than the garcinone B-ACE2 complex.

## 4. Materials and Methods

### 4.1. Molecular Docking Study

#### 4.1.1. Ligand Preparation

All xanthone isolate molecules were prepared by drawing their 2D structures using ChemDraw 15.0. The molecules of chloroquine and α-mangostin were acquired from the PubChem database (https://pubchem.ncbi.nlm.nih.gov, accessed on 18 May 2023). All ligands were optimized into their native 3D conformations through MM2 (molecular mechanic 2) energy minimization using Chem3D 15.0, and the files were saved in PDB file format. Hydrogen atoms were corrected and Gasteiger charges were added to all ligands using AutoDockTools 1.5.6, and the files were saved in PDBQT file format [61,62].

#### 4.1.2. Receptor Preparation

The ACE2 (angiotensin-converting enzyme 2) structure was obtained from the crystal structure of the complex between ACE2 bound to the binding domain of SARS-CoV-2’s spike protein (PDB ID: 6M0J) [42]. The structure of Mpro (main protease) was obtained from the crystal structure of the complex between SARS-CoV-2’s main protease and the compound N3 (PDB ID: 6LU7) [47]. Receptor structures were extracted from the complex using Biovia Discovery Studio Visualizer v21.1.0.20298 and saved in PDB file format [63]. The receptors were prepared by adding hydrogen atoms and Kollman charge parameters using AutoDockTools 1.5.6, and the prepared receptor was saved as a PDBQT file [64].

#### 4.1.3. Molecular Docking Simulation

All ligands were docked to ACE2 and Mpro using AutoDock 4.2.6 version. Grid parameters were created with the grid box directed to the binding region of ACE2 (X: −36.126, Y: 32.573, and Z: 3.383) and Mpro (X: −9.732, Y: 11.403, and Z: 68.925) [27,65]. Lamarckian GA (genetic algorithm) was utilized in preparing docking parameters. The docking results for each ligand were then evaluated based on the lower binding energy (ΔG value) of most clusters, inhibition constant (Ki), and visualization of amino acid residues interaction using Biovia Discovery Studio Visualizer v21.1.0.20298 [64].

### 4.2. Lipinski’s Rule of Five Filtration

All xanthone isolates were evaluated for oral administration suitability using Lipinski’s rule of five filtration. The rules include molecular weight (MW) ≤ 500 Da, hydrogen bond donor ≤ 5, hydrogen bond acceptor ≤ 10, and LogP ≤ 5 [52]. This evaluation was performed in admetSAR webserver (http://lmmd.ecust.edu.cn/admetsar2, accessed on 18 May 2023) [66].

### 4.3. Pharmacokinetic Profile and Toxicity Prediction

Pharmacokinetic profiles were predicted, including absorption, distribution, and metabolism. admetSAR webserver (http://lmmd.ecust.edu.cn/admetsar2, accessed on 19 May 2023) was used for this evaluation [66]. The absorption parameter was represented by human intestinal absorption (HIA) and caco-2 cell permeation, the distribution parameter was represented by protein plasma binding (PPB) and blood–brain barrier (BBB), metabolism parameter was represented by CYP (cytochrome P 450) inhibitor or substrate suitability, and toxicity was evaluated through carcinogenicity and Ames test for mutagenicity [67].

### 4.4. Molecular Dynamic Simulation

This simulation was accomplished to assess the interaction stability of the xanthone isolate with the best docked, the most fit of Lipinski’s rule, as well as the most suitable pharmacokinetic prediction. The evaluation was executed using the iMODS webserver (https://imods.iqfr.csic.es, accessed on 21 May 2023) [37]. The stability of interaction was measured through deformability, mobility profiles (B-factor), eigenvalues, variance, co-variance map, and elastic network [68].

## 5. Conclusions

The computational study revealed that xanthone isolates other than α-mangostin have high potential as anti-SARS-CoV-2 in the treatment of COVID-19 through the mechanism of ACE2 and Mpro inhibition. Among all isolates, only garcinone B, which apart from having good inhibitory potential, also exhibits good drug-likeness and toxicity profile. The interaction between garcinone B with ACE2 and Mpro is indicated to be stable based on molecular dynamic studies, represented by its higher eigenvalue. Thus, garcinone B could be a promising candidate for analysis in further studies regarding its potential in COVID-19 treatment.

## Figures and Tables

**Table 1:** Parameters obtained presenting molecular docking results against ACE-2.

Compound	ΔG (kcal/mol)	Ki (μM)	Amino Acid Residues Interaction		
			Hydrogen Bond	Hydrophobic	Others
Chloroquine	−4.1	991.4	Lys31	π-π stacked: His34; Alkyl: His34; π-alkyl: Lys31, His34	N/A
Remdesivir	−4.38	611.23	Lys31, Glu35	π-alkyl: His34	N/A
α-Mangostin	−4.27	746.2	Asp30	π-π stacked: His34; π-alkyl: Lys31, His34	π-anion: Asp30
7-*O*-demethyl mangostanin	−5.17	162.05	Asp30, Lys31, Glu35	π-π stacked: His34; Amide-π stacked: His34; π-alkyl: Lys31, His34	π-anion: Asp30
Mangostanin	−4.35	653.11	Lys31	π-π stacked: His34; Alkyl: His34; π-alkyl: Lys31, His34	π-anion: Asp30
8-Deoxygartanin	−5.13	173.07	Lys31, Glu35	π-π stacked: His34; Alkyl: Lys31	π-sigma: His34
Gartanin	−5.13	174.42	N/A	π-π stacked: His34; Amide-π stacked: His34; π-alkyl: Lys31, His34	van der Waals: Glu35
Garcinone E	−3.65	2130	Thr27	π-alkyl: Lys31	N/A
Trapezifolixanthone	−6.01	39.34	Asp30, Glu35	π-π stacked: His34; Amide-π stacked: His34; Alkyl: His34; π-alkyl: Lys31, His34	Salt bridge: Lys31; π-sigma: His34
Padiaxanthone	−5.75	61.41	Asp30, Lys31	π-π stacked: His34; Amide-π stacked: His34; Alkyl: His34; π-alkyl: Lys31, His34	π-anion: Asp30; van der Waals: Glu35
Tovophyllin A	−4.08	1020	Asp30, Lys31	π-π stacked: His34; Alkyl: His34; π-alkyl: Lys31, His34	N/A
1,5,8-Trihydroxy-3-methoxy-2-prenylxanthone	−5.17	161.09	N/A	π-π stacked: His34; Alkyl: His34; π-alkyl: Lys31, His34	N/A
Garcinone B	−5.21	152.73	Asp30, Glu35	π-π stacked: His34; Alkyl: His34; π-alkyl: Lys31, His34	N/A
1,7-Dihydroxy-2-(3-methylbut-2-enyl)-3-methoxyxanthone	−4.61	418	Lys31, Glu35	Alkyl: His34; π-alkyl: Lys31, His34	π-sigma: His34
Mangostenone D	−5.33	122.96	Thr27	π-π stacked: His34; Alkyl: His34; π-alkyl: Lys31, His34	π-anion: Asp30
Mangostinone	−6.81	10.19	Glu35	π-π stacked: His34; Alkyl: His34; π-alkyl: Lys31, His34	Attractive charge: Lys31
1,7-Dihydroxy-2-(3-methylbut-2-enyl)-3-methoxyxanthone	−4.85	279.52	Lys31	Amide-π stacked: His34; Alkyl: His34; π-alkyl: Lys31, His34	van der Waals: Glu35

**Table 2:** Parameters obtained presenting molecular docking results against Mpro.

Compound	ΔG (kcal/mol)	Ki (μM)	Amino Acid Residues Interaction		
			Hydrogen Bond	Hydrophobic	Others
Chloroquine	−7.11	6.10	His164	Alkyl: His163, His172, Arg188	π-sigma: His41; π-sulfur: Cys145
Remdesivir	−6.50	17.33	Glu166, Thr190, Gln192	Alkyl: Pro168	N/A
α-Mangostin	−8.31	0.805	Cys145, Glu166, Thr190, Gln192	Alkyl: His163, His172; π-alkyl: Met165	π-π T-shaped: His41
7-*O*-demethyl mangostanin	−8.97	0.268	Thr190, Gln192	Alkyl: Cys145, His163; π-alkyl: Met165	π-π T-shaped: His41; π-sigma: Gln189
Mangostanin	−7.92	1.57	Glu166, Arg188	Alkyl: His41, Met49, Cys145, His163, Pro168; π-alkyl: Met165	N/A
8-Deoxygartanin	−8.87	0.318	Met49, Glu166	Alkyl: His163, His172; π-alkyl: Cys145	N/A
Gartanin	−9.13	0.203	His163, Glu166, Thr190	Alkyl: Pro168; π-alkyl: Met165	N/A
Garcinone E	−9.61	0.091	His164, Met165, Arg188	Alkyl: Cys44, Met49, Cys145, His163	π-π T-shaped: His41
Trapezifolixanthone	−9.34	0.143	Cys145, His164, Thr190, Gln192	Alkyl: His41, Met49; π-alkyl: Met165	π-lone pair: Glu166
Padiaxanthone	−10.23	0.032	Gly143, Glu166	Alkyl: Met49, His163, His172; π-alkyl: Cys145	π-sigma: His41
Tovophyllin A	−9.24	0.170	Met165, Arg188, Thr190, Gln192	Alkyl: His41, Met49, Pro52, His163; π-alkyl: Pro168	π-lone pair: Glu166
1,5,8-Trihydroxy-3-methoxy-2-prenylxanthone	−9.25	0.166	N/A	Alkyl: Pro52, Tyr54; π-alkyl: Met49, Met165	N/A
Garcinone B	−9.59	0.094	Leu141, Gly143, Ser144	Alkyl: Leu27, Cys145, His163; π-alkyl: His41, Met49, Met165	N/A
1,7-Dihydroxy-2-(3-methylbut-2-enyl)-3-methoxyxanthone	−7.24	4.90	Cys145, His164, Glu166	Alkyl: Met165	π-sulfur: Cys145
Mangostenone D	−8.56	0.528	Thr190	Alkyl: Cys145, His163, Pro168	π-sulfur: Met165
Mangostinone	−10.30	0.028	Cys145, His164, Thr190	Alkyl: His41, Met49, Arg188; π-alkyl: Met165	π-lone pair: Glu166
1,7-Dihydroxy-2-(3-methylbut-2-enyl)-3-methoxyxanthone	−9.98	0.048	Cys145, His164, Glu166	Alkyl: Leu27; π-alkyl: Met165	π-cation: His163; π-sigma: His41

**Table 3:** Lipinski’s rule of five characteristics of xanthone isolates.

Compound	Molecular Weight (g/mol)	Hydrogen Bond Donor	Hydrogen Bond Acceptor	LogP	Violation
7-*O*-demethyl mangostanin	380.44	3	5	5.08	1 (LogP > 5)
Mangostanin	342.35	3	6	3.58	0
8-Deoxygartanin	326.35	2	5	3.87	0
Gartanin	394.42	3	6	4.76	0
Garcinone E	380.44	3	5	5.08	1 (LogP > 5)
Trapezifolixanthone	394.42	3	6	4.76	0
Padiaxanthone	464.56	4	6	6.29	1 (LogP > 5)
Tovophyllin A	396.44	4	6	4.79	0
1,5,8-Trihydroxy-3-methoxy-2-prenylxanthone	408.45	2	6	5.06	1 (LogP > 5)
Garcinone B	396.44	3	6	4.68	0
1,7-Dihydroxy-2-(3-methylbut-2-enyl)-3-methoxyxanthone	380.44	3	5	5.30	1 (LogP > 5)
Mangostenone D	392.41	2	6	4.73	0
Mangostinone	462.54	3	6	6.26	1 (LogP > 5)
1,7-Dihydroxy-2-(3-methylbut-2-enyl)-3-methoxyxanthone	378.42	2	5	5.05	1 (LogP > 5)

**Table 4:** Pharmacokinetic profiles and toxicity of xanthone isolates predicted by admetSAR webserver.

Compound	Absorption		Distribution		Metabolism ^*)^		Toxicity ^*)^	
	HIA	Caco-2	PPB	BBB	Inhibitor	Substrate	Carcinogenicity	Ames Test (Mutagenicity)
7-*O*-demethyl mangostanin	0.9895	0.5775	0.525	0.915	+	−	+	−
Mangostanin	0.9886	0.4906	0.500	0.86	+	−	+	−
8-Deoxygartanin	0.9919	0.7836	0.500	0.933	+	−	+	−
Gartanin	0.9732	0.5446	0.625	0.733	+	−	+	−
Garcinone E	0.9855	0.4877	0.575	0.961	+	−	+	−
Trapezifolixanthone	0.9846	0.5797	0.600	0.779	+	−	+	−
Padiaxanthone	0.9465	0.7296	0.650	0.756	+	−	+	−
Tovophyllin A	0.9855	0.6397	0.575	0.769	+	−	+	−
1,5,8-Trihydroxy-3-methoxy-2-prenylxanthone	0.9743	0.7549	0.525	0.719	+	−	+	−
Garcinone B	0.9632	0.6002	0.55	0.708	+	−	−	−
1,7-Dihydroxy-2-(3-methylbut-2-enyl)-3-methoxyxanthone	0.9776	0.8016	0.525	0.946	+	−	+	−
Mangostenone D	0.9838	0.5834	0.675	0.823	+	−	+	−
Mangostinone	0.9846	0.6456	0.6	0.737	+	−	+	−
1,7-Dihydroxy-2-(3-methylbut-2-enyl)-3-methoxyxanthone	0.9905	0.592	0.575	0.921	+	−	+	−

*) (+) and (−) sign indicate the positive and negative results, respectively, of the predictions performed.

## Data Availability

All data generated or analyzed during this study are included in this published article.

## Sample Availability

Samples of the compounds are not available from the authors.
